# Preservation of the Sterile Matrix, Hyponychium, and Fingertip Pad in Fingertip Reconstruction With Composite Fingertip and Nail Bed Graft and Volar V-Y Advancement Flap

**Published:** 2017-09-08

**Authors:** Joshua T. Henderson, Steven A. Schulz, Andrew M. Swiergosz, Andrea R. Hiller, J. Stephen Gunn, Joshua H. Choo, Morton L. Kasdan, Bradon J. Wilhelmi

**Affiliations:** ^a^University of Louisville School of Medicine, Louisville, Kentucky; ^b^Division of Plastic and Reconstructive Surgery, Department of Surgery, University of Louisville, Louisville, Kentucky

**Keywords:** fingertip injury, composite graft, hyponychium, hook nail, advancement flap

## Abstract

**Background:** The goals of fingertip reconstruction are to achieve adequate soft-tissue coverage and a functional nail plate and to maintain sensation, proprioception, and cosmesis. **Objective:** We present a composite tissue graft and volar V-Y advancement flap for reconstruction of a traumatic amputation of a fingertip, which provided optimal preservation of the hyponychium and the volar pad for prevention of a hook nail. Historically, composite fingertip grafts have not been recommended for adults with large defects. **Methods:** The amputated nail bed, hyponychium, and a 10 × 20-mm segment of the fingertip were utilized as a composite graft for reconstruction of the nail bed in an adult. The addition of a volar V-Y advancement flap to reconstruct the fingertip was necessary for complete soft-tissue reconstruction. **Results:** The reconstruction resulted in nail plate adhesion without significant nail deformity and a functional and sensate fingertip. **Conclusion:** Components of amputated fingertips including the sterile matrix, hyponychium, and part of the fingertip can be utilized in a composite graft to yield satisfactory functional and cosmetic results in adults.

The goals of fingertip reconstruction are to achieve adequate soft-tissue coverage and a functional nail plate and to maintain sensation, proprioception, and cosmesis. The technique selected typically depends on the extent of the injury and experience of the surgeon. Skin grafts, local flaps, and open healing can suffice for less severe injuries, but injuries that result in significant nail bed and pulp deficits require advancement flaps or even distant flaps.^1^ Optimal aesthetic appearance may not be achieved with these flaps because of hyponychial tissue loss and subsequent absence of the volar support of the nail plate. Historically, composite fingertip grafts have not been recommended for adults with large defects, as revascularization was thought to be unreliable.^2^^-^5 In adults with transverse or dorsal oblique planes of fingertip amputation, a composite nail bed graft that includes the sterile matrix, hyponychium, and part of the fingertip can be utilized with a volar V-Y advancement flap to provide functional reconstruction with improved cosmetic results.

## METHODS

**Figure 1:**
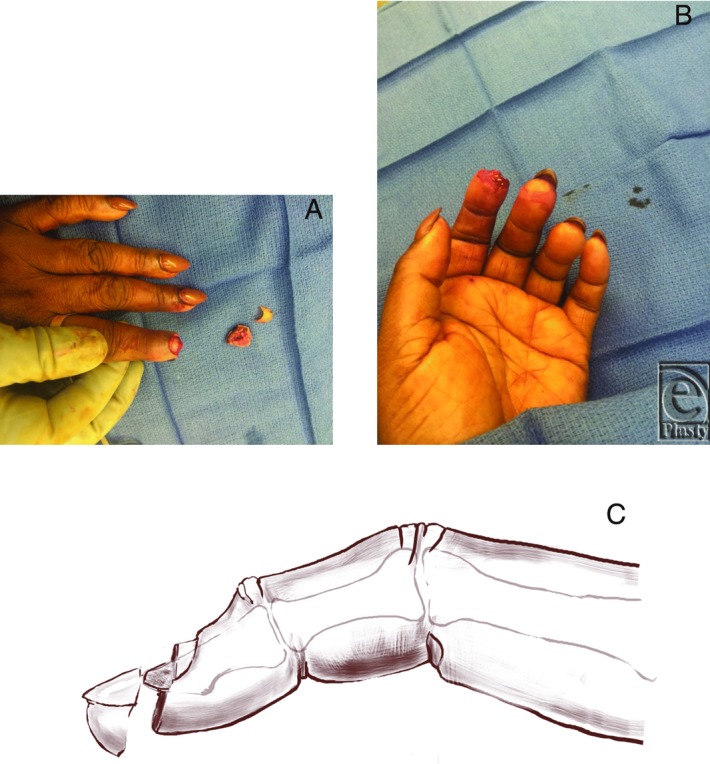
The fingertip injury showing the transverse plane of amputation and loss of the nail bed, hyponychium, and pulp (a, b). Sketch of the lateral view of the amputated segment and transverse plane of injury of the finger (c).

A 55-year-old African American woman presented to the emergency department after sustaining a crush amputation of her right index fingertip in a door jam. She had a single avulsed segment of the distal half of the nail bed, nail plate, hyponychium, and fingertip pad (Figs 1*a*-1*c*).

**Figure 2:**
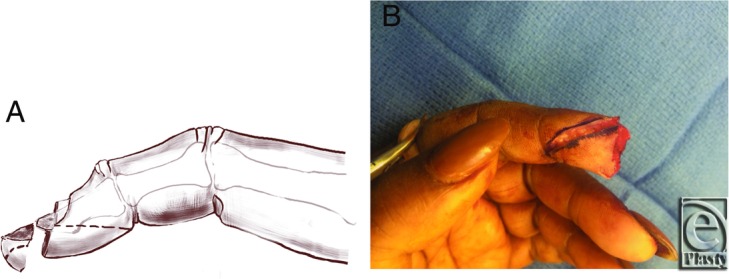
Sketch of the lateral view of the finger and amputated segment marked prior to incision and mobilization of the volar V-Y advancement flap (a). Mobilization of the volar V-Y advancement flap (b). Insetting and light approximation with few sutures followed mobilization.

**Figure 3:**
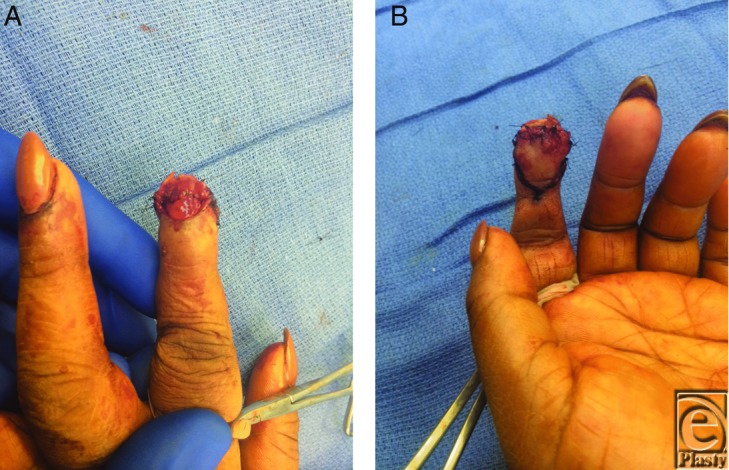
The debrided amputated segment of sterile matrix, hyponychium, and part of the fingertip were utilized as a composite graft and repaired to the injured fingertip. The reconstructed nail bed (a) and the debrided amputated fingertip were sutured to the volar V-Y advancement flap (b).

Radiographs did not document a fracture. The patient was concerned about the final appearance. The extent of soft-tissue loss from the amputation of the distal tip limited local flap options to completely cover the defect. A volar V-Y advancement flap was performed to support and replace the missing fingertip pad (Figs 2*a* and 2*b*). Some of the amputated fingertip pad and nail bed were crushed and debrided. This resulted in a deficit of the volar pulp, but approximately half of the sterile matrix was not involved. This debridement was necessary to improve reliability of the composite graft for this adult who recently quit smoking. The amputated portion was utilized as a composite graft of sterile matrix, hyponychium, and a 10 × 20-mm segment of the fingertip. The composite graft was sutured to the distal V-Y advancement flap for complete coverage of the distal phalanx and to maintain adequate volar support of the nail plate (Figs 3*a* and 3*b*).

## RESULTS

**Figure 4:**
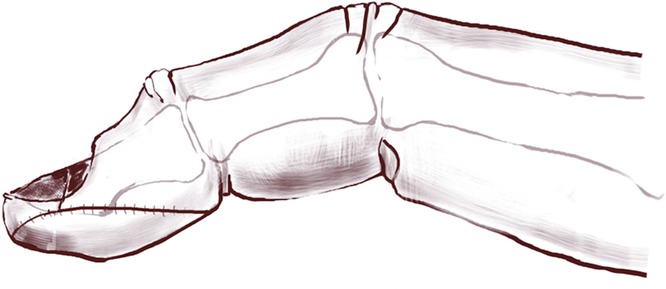
Sketch of the lateral view of the composite graft sutured to the volar V-Y advancement flap.

**Figure 5:**
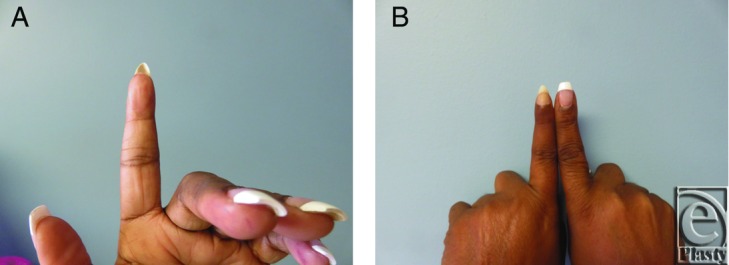
Postoperative photographs at 10-month follow-up (a, b). When compared with the contralateral index finger, there was some atrophy of the distal phalanx (b). The patient was satisfied with the function, sensation, and appearance of her reconstructed fingertip.

The composite graft took successfully, and the volar V-Y advancement flap provided volar support (Fig 4). The composite graft of sterile matrix, hyponychium and part of the fingertip resulted in nail plate adherence without a hook nail deformity at 10-month follow-up (Figs 5*a* and 5*b*). Although there was some expected atrophy of the distal phalanx, she did not have any complications, painful scarring, hypoesthesia, or hook nail deformity. The patient was satisfied with the function, sensation, and appearance of her reconstructed fingertip.

## DISCUSSION

The main goals in the reconstruction of complex fingertip and nail bed injuries are to achieve nontender coverage and preserve protective sensation. An ideal cosmetic outcome should be sought, as the fingertip is one of the most exposed areas of the body. Minimizing nail plate abnormalities has both functional and aesthetic advantages when reconstructing fingertip injuries. Hook nail deformities result from the loss of volar bony and soft-tissue support, thus the nail curves and forms a tender tip.^6^^-^8 Hook nail deformities also significantly diminish fingertip aesthetics. Incorporating the hyponychium in a composite graft can significantly bolster the volar fingertip pad, aiding in the prevention of a hook nail deformity, and provide a mechanical and immunologic barrier against perionychial infection.^6^^,^^7^

When bone or tendon is exposed at the base of a fingertip wound, a flap is typically preferred for reconstruction.^4^ Local flap options include the volar V-Y advancement flap, modified volar V-Y advancement “cup” flap, bilateral V-Y advancement flap, oblique triangular island flap, volar rectangular advancement flap (used mostly for reconstruction of the thumb), and homodigital island flaps.^9^^-^14 The orientation and configuration of the wound and the injured digit influence which flap is best for a given fingertip injury.^4^ A volar V-Y advancement flap is appropriate for injuries occurring in the dorsal oblique and transverse planes.^9^ Mobilization of glabrous-like tissue retains sensation in the reconstructed fingertip.^9^

The patient presented with a type 2 fingertip injury according to the Allen classification.^15^ The transverse plane of injury made the volar V-Y advancement flap a suitable option for reconstruction, but this flap was not adequate by itself due to loss of the hyponychium and distal nail bed with the amputated segment. Were the amputated segment not utilized as a composite graft, a hook nail deformity or loss of nail would be likely, as a hook nail deformity results from loss of support of the distal nail bed over the distal phalanx.^6^^-^8 The treatment and prevention of hook nail deformities with advancement flaps have been reported.^16^^,^^17^ Still, consideration should be given to utilization of the amputated tissue as a composite graft to bolster the volar support when local flaps are inadequate.

Composite grafting of the fingertip and the nail bed is a technique used in children but generally discouraged in adults because of inadequate revascularization.^1^^-^4 The composite graft in adults is thought to not revascularize quickly enough because of metabolic demands of the hyponychium and the fingertip. Amputations occurring more distally on the fingertip and shorter lengths of time between injury and reattachment confer greater rates of composite graft survival.^2^^,^^3^^,^^5^ Retrospective studies evidence successful composite grafts in adult fingertip injuries, with complete graft survival mostly limited to clean-cut amputations distal to the nail base.^18^^-^21 There are also reports of favorable results when utilizing a composite fingertip graft with advancement flaps when necessary.^22^^-^26

Reports of composite fingertip grafts in conjunction with local flaps do not highlight the importance of the inclusion of the hyponychium with the graft.^18^^-^26 Preservation of the nail bed and sterile matrix with adequate support by the hyponychium is essential both for nail plate adherence and for prevention of hook nail deformities.^6^^-^8 Immunologically, the hyponychium supplies the graft and the reconstructed fingertip with an abundance of lymphocytes.^6^^,^^7^ These lymphocytes, in addition to the role of the hyponychium as a mechanical barrier at the edge of the nail bed, help prevent perionychial infection.^6^^,^^7^ The hyponychium's requirement for perfusion via plasma imbibition does not preclude graft survival in an adult.
